# Mechanical Properties of Aluminum Alloys under Low-Cycle Fatigue Loading

**DOI:** 10.3390/ma12132064

**Published:** 2019-06-27

**Authors:** Xuehang Zhao, Haifeng Li, Tong Chen, Bao’an Cao, Xia Li

**Affiliations:** 1 College of Civil Engineering, Huaqiao University, Xiamen 361021, China; 2 Key Laboratory for Structural Engineering and Disaster Prevention of Fujian Province, Xiamen 361021, China

**Keywords:** aluminum alloy, low-cycle fatigue loading, mechanical properties, material property test, finite element simulation

## Abstract

In this paper, the mechanical properties of 36 aluminum alloy specimens subjected to repeated tensile loading were tested. The failure characteristics, stress-strain hysteresis curves and its corresponding skeleton curves, stress cycle characteristics, and hysteretic energy of specimens were analyzed in detail. Furthermore, the finite element model of aluminum alloy specimens under low-cycle fatigue loading was established and compared with the experimental results. The effects of specimen parallel length, parallel diameter, and repeated loading patterns on the mechanical properties of aluminum alloys were discussed. The results show that when the specimen is monotonously stretched to fracture, the failure result from shearing break. When the specimen is repeatedly stretched to failure, the fracture of the specimen is a result of the combined action of tensile stress and plastic fatigue damage. The AA6061, AA7075, and AA6063 aluminum alloys all show cyclic softening characteristics under repeated loading. When the initial stress amplitude of repeated loading is greater than 2.5%, the repeated tensile loading has a detrimental effect on the deformability of the aluminum alloy. Finally, based on experiment research as well as the results of the numerical analysis, the calculation method for the tensile strength of aluminum alloys under low-cycle fatigue loading was proposed.

## 1. Introduction

Aluminum alloys have the advantages of low weight, high strength, ease of processing, low-temperature resistance, corrosion resistance, and low maintenance. They are widely used in machinery manufacturing, shipbuilding, aerospace, and chemical industries. In recent years, with the continuous promotion and application of fabricated buildings in the field of civil engineering, aluminum alloys have gradually become one of the most extensively used building materials for the main stressed members in construction engineering. Concerning the large-span aluminum alloy reticulated shell structures used in construction, Guo et al. [1,2] used theoretical analysis, experimental research, and numerical simulation methods to study the influence of semi-rigid joints on the mechanical properties of single-layer reticulated shells. Under the action of major earthquakes, the metal-stressed members of the main structure in the actual construction project are prone to low-cycle fatigue damage, and the failure mechanism is the plastic deformation of the material under low-cycle fatigue loading [3,4].

At present, scholars have carried out fatigue performance tests under low-cycle fatigue loading on different types of aluminum alloys, both at home and abroad. Shaha et al. and Huang et al. [5,6] conducted low-cycle fatigue tests on Al-Si casting alloys with Ti, V, and Zr, and studied their tensile and fatigue properties. The results showed that the tensile strength and fatigue life of the aluminum alloy can be improved by adding transition metal elements such as Ti, V, and Zr. Liu et al. and Lee et al. [7,8] conducted tensile tests and microstructure observations on friction-stir welded aluminum alloys to study the mechanical properties and evolution characteristics of fracture mechanisms. In order to study the low cycle fatigue characteristics, cyclic stress response characteristics and fracture behavior of aluminum alloys, Srivatsan et al. [9] conducted low cycle fatigue tests with the high purity aluminum alloy AA7150 under the strain control mode. Studies have shown that the AA7150 aluminum alloy exhibits evident cyclic softening phenomenon. Hao et al. [10] carried out fatigue tests on aluminum alloys under different loading patterns and discussed the effects of loading strain on the fatigue properties and microstructure characteristics of aluminum alloys. Arcari et al. [11,12] studied the stress relaxation of aluminum alloys and discussed the accuracy of different models for the mean stress fitting of aluminum alloys. Kim et al. and Lin et al. [13,14] conducted low-cycle fatigue tests on aluminum alloys in different environments to reveal the mechanism of the occurrence and development of corrosion fatigue cracks in aluminum alloys. Burns et al. and Cédric et al. [15,16] conducted tensile tests and microstructure observations on aluminum alloys to investigate the fatigue properties of aluminum alloys in low-temperature environments. Conley et al. and Wang et al. [17,18] proposed a mathematical model for predicting the size and distribution of micropores in order to research the effect of the casting defects of aluminum alloys on fatigue life. This model can investigate the effects of processing parameters such as cooling rate, hydrogen content, and grain refinement on micropore formation. Azadi and Shirazabad and Zhu et al. [19,20] carried out the fatigue performance test of the cast aluminum alloy subjected to different heat treatment processes. The research showed that the heat treatment process can effectively improve the mechanical properties and low cycle fatigue life of aluminum alloys, and the heat treatment conducted at high temperatures is conducive to the improvement of fatigue performance. Wang et al. [21] conducted a low cycle fatigue performance test on aluminum alloy buckling-restrained braces and proposed a low cycle fatigue damage evaluation formula for aluminum alloy buckling-restrained braces. Underhill and DuQuesnay and DuQuesnay and Underhill [22,23] conducted fatigue tested under differ spectra to study the fatigue properties and to determine if fatigue life might become bimodal. Zhang et al. [24] combined the critical plane principle, the microcosmic mechanism of fatigue damage, and the additional hardening of non-proportional loading, defined a new damage parameter.

In summary, different types of aluminum alloys are quite different in the mechanical properties including strength, ductility, and stiffness. At present, there are few studies on the mechanical properties of extensively used aluminum alloys in construction engineering. In this study, AA6061, AA7075, and AA6063 aluminum alloys, which are extensively used in construction engineering, were selected as research objects. The mechanical properties of these three types of aluminum alloys under low-cycle fatigue loading are discussed. The work schedule in this study is carried out as follows: (a) Carrying out the mechanical property testing of aluminum alloys under repeated tension, analyzing the failure characteristics, stress-strain hysteresis curves and its corresponding skeleton curves, stress cycle characteristics, and hysteretic energy of specimens. (b) Conducting numerical simulations of the material property tests under repeated tension and comparing with test results to verify the accuracy of the finite element model. (c) Analyzing the parameters of the mechanical properties of aluminum alloys under low-cycle fatigue loading and obtaining the influence of the main parameters on the mechanical properties of aluminum alloys. (d) Based on the results of experimental study and numerical analyses, the strength design formula of aluminum alloys is proposed to provide reference data for the application of aluminum alloys in seismic engineering.

## 2. Experimental Setup

**Figure 1:**
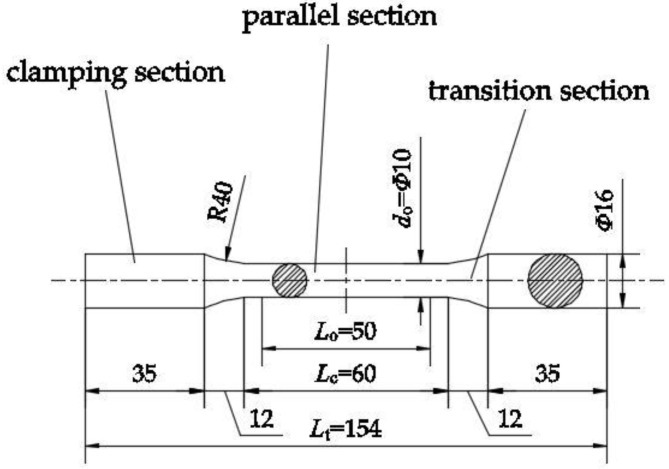
Design of specimens (Units: mm).

According to the ISO 6892-1: 2016 Metallic materials-Tensile testing-Part 1: Method of test at room temperature [25], specimen size was specified; the elastic modulus, yield strengths, and tensile strengths of specimens were calculated and analyzed. A total of 36 specimens were designed in this test, all of which were cylindrical specimens of the same size, as shown in Figure 1. The intermediate parallel section length *L*_c_ is 60 mm and the diameter *d*_o_ is 10 mm; the length of the clamping section at both ends is 35 mm, and the diameter is 16 mm; the radius *R* of the transition section is 40 mm. In this study, aluminum alloy specimens were directly processed by the use of the computer numerical control (CNC) grinding method for surface finishing, and the value of surface roughness (*Ra*) of the specimens is 0.5 μm. Based on the material type of the aluminum alloys, the specimens were divided into three groups. The design parameters of the specimens are shown in Table 1. In the specimen number, the letter A stands for AA6061 aluminum alloy, the letter B stands for AA7075 aluminum alloy, and the letter C stands for AA6063 aluminum alloy. The number following the specimen number corresponds to the particular load mode number of X. The first figure stands different stress amplitudes of repeated loading; The second figure stands different numbers of loading cycles.

**Figure 2:**
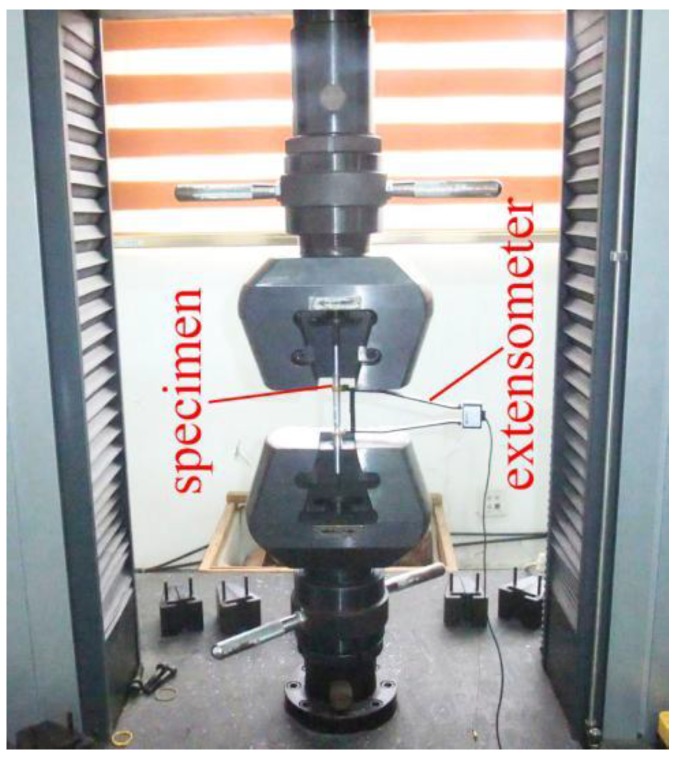
Loading device.

**Figure 3:**
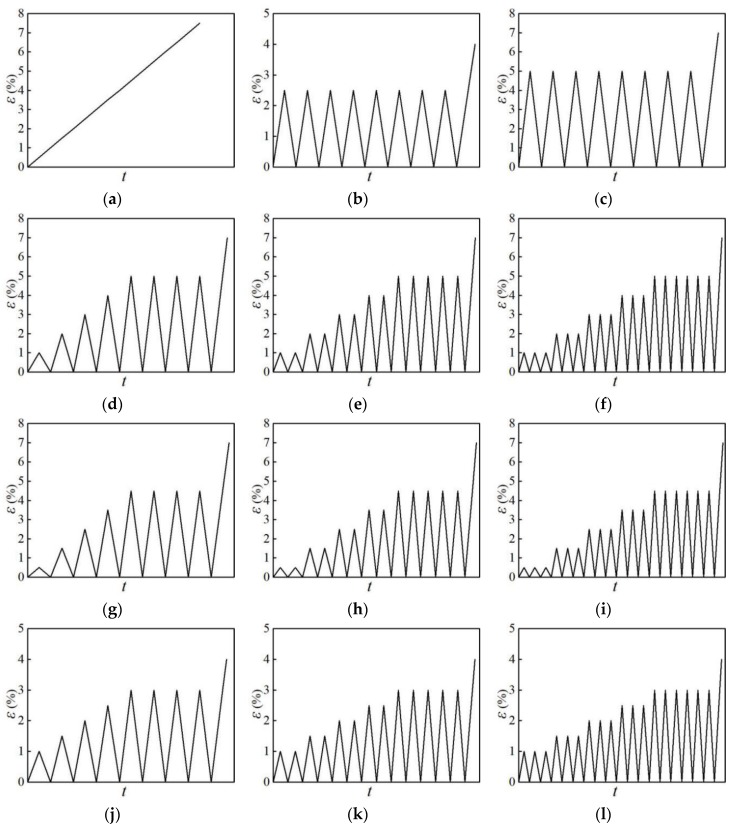
Loading patterns of specimens (Units: s): (**a**) loading pattern NM-1; (**b**) loading pattern NM-2-1; (**c**) loading pattern NM-2-2; (**d**) loading pattern NM-3-1; (**e**) loading pattern NM-3-2; (**f**) loading pattern NM-3-3; (**g**) loading pattern NM-4-1; (**h**) loading pattern NM-4-2; (**i**) loading pattern NM-4-3; (**j**) loading pattern NM-5-1; (**k**) loading pattern NM-5-2; (**l**) loading pattern NM-5-3.

The experiment was carried out using a CMT5105 electronic universal testing machine at the Structural Laboratory of the Huaqiao University (Xiamen, China). The loading device is shown in Figure 2. The strain of the specimen was measured by a tension-compression extensometer (MTS Systems (China) Co., Ltd., Shenzhen, China). The gauge length of the extensometer was 50 mm, and the original gauge length *L*_0_ was 50 mm; the tension and pressure ranges of the extensometer were both 25%. A total of 12 loading patterns were used in the test, and the loading process was process controlled by the displacement; the test loading speed was 0.8 mm/min. The loading scheme is shown in Figure 3. 

## 3. Results and Discussion 

### 3.1. Fracture Features

**Figure 4:**
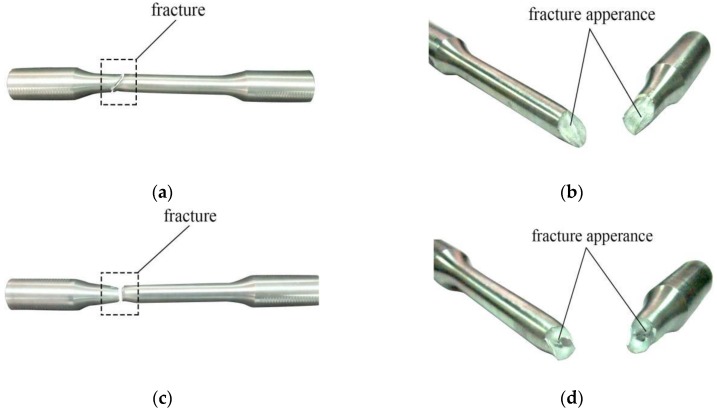
Fracture features of specimens: (**a**) B-1 specimen; (**b**) fracture appearance of B-1 specimen; (**c**) B-3-1 specimen; (**d**) fracture appearance of B-3-1 specimen.

Specimens form different fracture morphologies under uniaxial tension and repeated loading, that is, two failure modes are produced. When the specimen is monotonously stretched to breaking, the fracture of the specimen is tensile, which is the first failure mode, as shown in Figure 4a,b. The color of the fracture is dark gray and shows irregular fibers, and the surface of the fracture is rough; the cross section of the fracture is 45° from the direction of the tensile stress, which indicates a shear fracture.

When the specimen is subjected to repeated tension, it produced fatigue damage, as shown in Figure 4c,d. Because the loading scheme of this test has limited number of cycles and a small stress amplitude, the second failure mode is a mixed fracture having both tensile and fatigue components. The fracture surface is dark gray and uneven, the inner ring of the fracture cross section is perpendicular to the tensile stress, and the outer ring is at an angle of 45° with the direction of the tensile stress. The failure of the specimen is the result of the combined action of tensile stress and plastic fatigue damage. 

### 3.2. Stress-Strain Curves

The stress-strain curves of the specimens were extracted, and the influences of material type and loading pattern on the mechanical properties of the specimen were analyzed. In this paper, σ is the force at any moment during the test divided by the original cross-sectional area.

#### 3.2.1. Influence of Material Type

**Figure 5:**
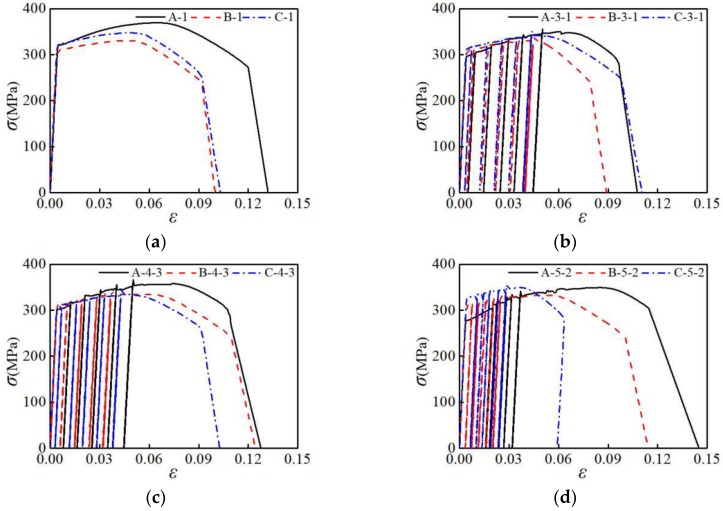
Influence of material type on stress-strain curve: (**a**) loading pattern NM-1; (**b**) loading pattern NM-3-1; (**c**) loading pattern NM-4-3; (**d**) loading pattern NM-5-2.

The stress-strain curves of three different types of aluminum alloy specimens under loading patterns NM1, NM3-1, NM4-3, and NM5-2 were selected for comparative analysis, as shown in Figure 5. The specimens of three different material types all have no obvious yielding platform both under uniaxial tension and repeated loading. In the elastic deformation stage, the deformation curves of the three sets of specimens are coincident, indicating that the elastic modulus of the three types of aluminum alloys are the same. As shown in Figure 5b–d, the stress-strain curve when the specimen is unloaded is substantially parallel to that in the elastic phase. Under the four loading patterns, the maximum tensile stress of group A is greater than those of groups B and C; and the tensile strain corresponding to the maximum tensile stress of group A is also larger. It shows that the tensile strength of AA6061 aluminum alloy is greater than that of AA7075 and AA6063 aluminum alloys. The AA6061 aluminum alloy reaches its maximum tensile strength later. 

#### 3.2.2. Influence of Loading Pattern

**Figure 6:**
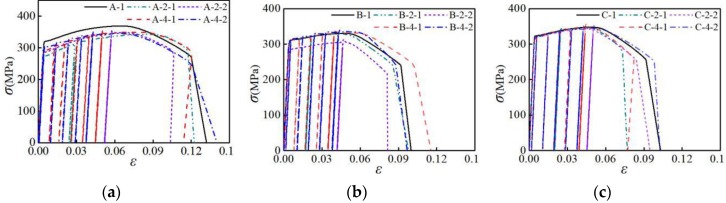
Influence of different loading pattern on stress-strain curve: (**a**) group A of specimens; (**b**) group B of specimens; (**c**) group C of specimens.

The stress-strain curves of the three groups were selected and compared under the partial loading pattern, as shown in Figure 6. Under the NM4-1 and NM4-2 loading patterns, with the increase in the number of loading cycles, and the tensile strength of the specimen changes a little. This indicates that the number of loading cycles has little effect on the tensile strength of all the three types of aluminum alloys. Under the NM2-1 and NM2-2 loading patterns, the ultimate strain values of the three groups of specimens are all decreased. It is demonstrated that when the initial stress amplitude of repeated loading is greater than 2.5%, the repeated tensile loading has a detrimental effect on the deformability of the aluminum alloy.

### 3.3. Stress Cycle Characteristics

**Figure 7:**
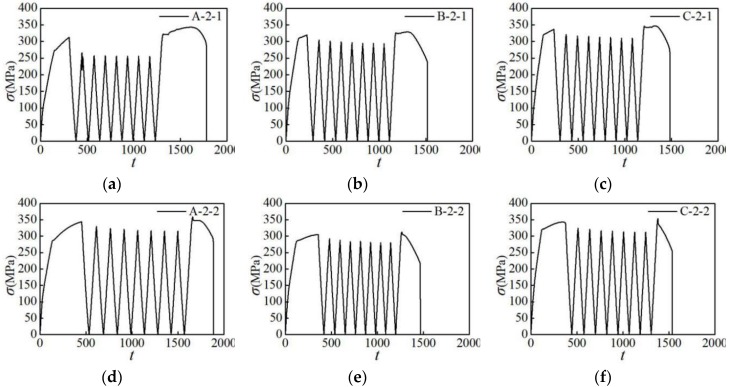
Stress-time curves of specimens (Units: s): (**a**) A-2-1 specimen; (**b**) B-2-1 specimen; (**c**) C-2-1 specimen; (**d**) A-2-2 specimen; (**e**) B-2-2 specimen; (**f**) C-2-2 specimen.

In order to analyze the stress cycle characteristics of aluminum alloys during repeated loading, the stress-time curves of three groups of specimens under the partial loading pattern were extracted and analyzed, as shown in Figure 7. It can be seen from Figure 7 that during the repeated loading process, the stresses in the three groups of specimens all decrease with the increase in the number of loading cycles, showing a considerable cyclic softening characteristic. In the initial stage of repeated loading, the stress decreases sharply with the increase in the number of loading cycles, and the material shows rapid cyclic softening, while in the later stage of repeated loading, the stress drop of the specimen gradually slows down. It is indicated that the AA6061, AA7075, and AA6063 aluminum alloys all have cyclic softening characteristics under repeated loading, that is, they show rapid softening in the initial stage of repeated loading, while the softening rate reduces in the later stage.

### 3.4. Hysteresis Energy

The test results of the maximum tensile stress *f*_u_ and its corresponding strain *ε*_u_, hysteresis energy *E*, and elongation rate *δ* are extracted, as summarized in Table 2.

#### 3.4.1. Influence of Material Type

**Figure 8:**
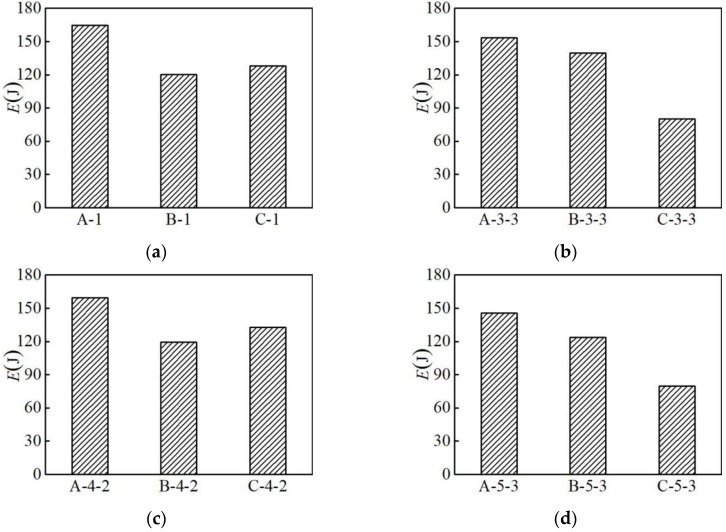
Influence of material type on hysteresis energy: (**a**) loading pattern NM-1; (**b**) loading pattern NM-3-3; (**c**) loading pattern NM-4-2; (**d**) loading pattern NM-5-3.

The hysteretic energy diagrams of three different types of aluminum alloy specimens in the partial loading pattern are selected for analysis, as shown in Figure 8. Under the NM1, NM3-3, NM4-2, and NM5-3 loading patterns, the hysteretic energy of group A is greater than those of groups B and C. It shows that the energy dissipation capacity of the AA6061 aluminum alloy is better than the AA7075 and AA6063 aluminum alloys. 

#### 3.4.2. Influence of Loading Pattern

**Figure 9:**
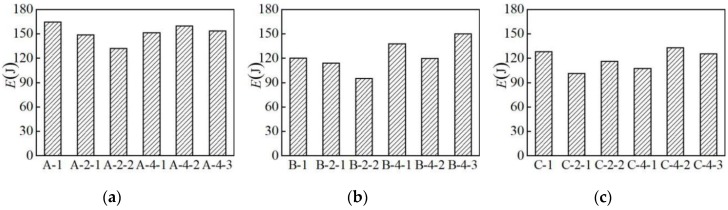
Influence of different loading pattern on hysteresis energy: (**a**) group A of specimens; (**b**) group B of specimens; (**c**) group C of specimens.

The hysteretic energy maps of the three groups of specimens under different loading patterns are selected for analysis, as shown in Figure 9. It is indicated that the number of loading cycles has little effect on the hysteretic energy. Compared with uniaxial tension, the hysteresis energy of all the three groups of specimens decreases under the NM2-1 and NM2-2 loading patterns. This indicates that when the initial stress amplitude of repeated loading is higher than 2.5%, the repeated tensile loading will induce plastic damage and reduce energy dissipation capacity.

## 4. Finite Element Model and Parameter Analysis

### 4.1. Finite Element Model and Verification

**Figure 10:**
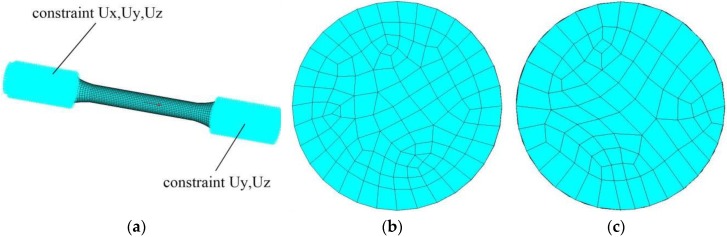
Finite element model: (**a**) boundary conditions of the model; (**b**) mesh generation of parallel section; (**c**) mesh generation of clamping section.

**Figure 11:**
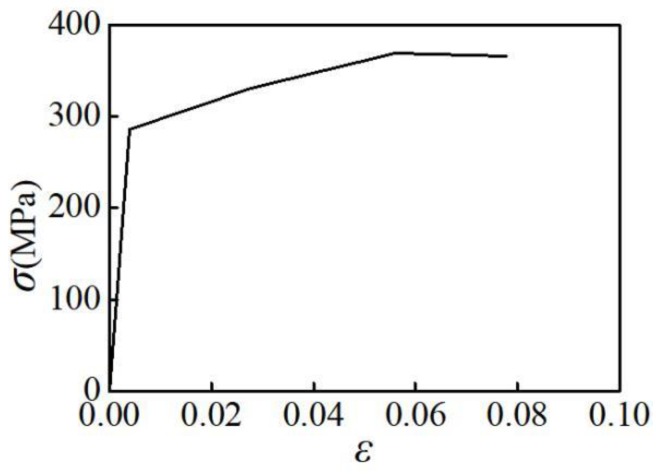
Stress-strain curve of aluminum alloy.

Based on the design parameters of the aluminum alloys, ANSYS 16.0 is used to establish the finite element model of the specimen. The finite element model is simulated by the eight-node hexahedral solid element SOLID185. Meshing directly affects analysis speed and accuracy of analysis results in finite element simulation. Through adjusting on computer, the element size of intermediate parallel section is 1 mm; the element size of transition section and clamping section is 1.5 mm, 15372 elements can be generated by sweeping. A line displacement constraint is applied to the clamping sections at both ends, and the junction of the upper clamping section is applied according to the loading displacement corresponding to the loading strain. The finite element model is shown in Figure 10. According to density, elasticity modulus and Poisson's ratio of the aluminum alloy materials, the multilinear hardening model (KINH) in ANSYS is used to define the constitutive relationship of the aluminum alloys. Referring to the yield points, maximum stress points, breaking points of the stress-strain curves plotted by applying uniaxial tension, the constitutive relationship of the aluminum alloy in the finite element model is defined, as shown in Figure 11.

**Figure 12:**
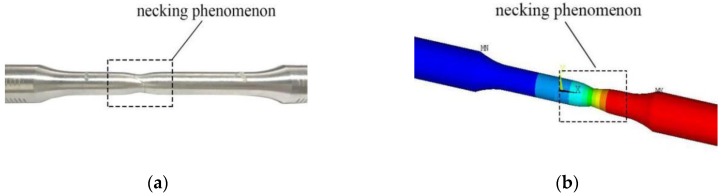
Failure morphology of specimen and simulation: (**a**) failure morphology of specimen; (**b**) failure morphology of finite element simulation.

The failure morphology and finite element simulation results of the B-3-3 specimen are extracted as shown in Figure 12. As can be seen from Figure 12, the deformation characteristics of the two methods in the parallel section agree well with each other, and the finite element model can simulate the necking phenomenon of the specimen. It shows that the finite element model can accurately simulate the plastic deformation characteristics of the aluminum alloy specimens.

**Figure 13:**
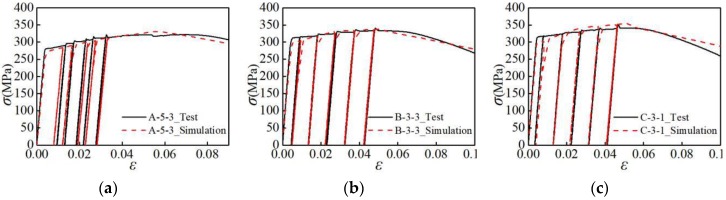
Stress-strain curves of specimens and simulation: (**a**) A-5-3 specimen; (**b**) B-3-3 specimen; (**c**) C-3-1 specimen.

To further verify the accuracy of the finite element model, the stress-strain curves of the three groups of specimens are compared, as shown in Figure 13. As can be seen from the figure, the strength, stiffness, and envelope area of the finite element simulation curve agree well with the curve from the experiment. Thus, the finite element model developed in this study is highly accurate.

### 4.2. Numerical Analysis Specimens

**Figure 14:**
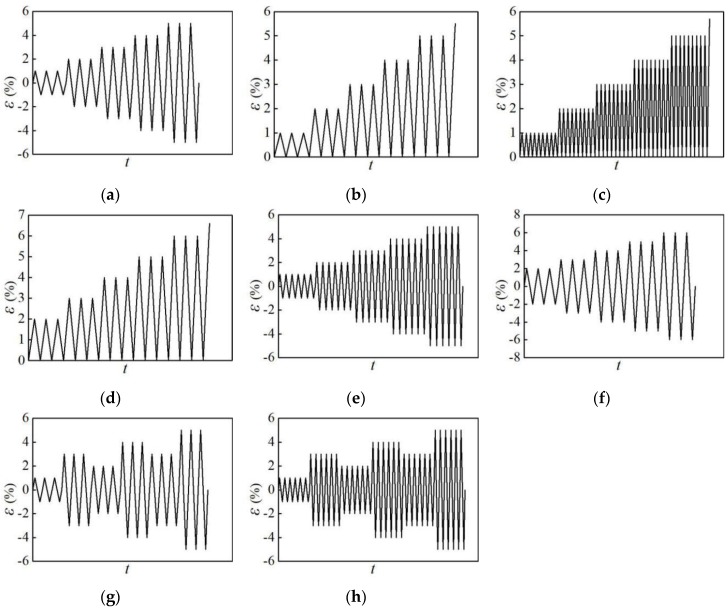
Loading patterns of specimens for the numerical analysis (Units: s): (**a**) loading pattern NM-6; (**b**) loading pattern NM-7-1; (**c**) loading pattern NM-7-2; (**d**) loading pattern NM-7-3; (**e**) loading pattern NM-8-1; (**f**) loading pattern NM-8-2; (**g**) loading pattern NM-9-1; (**h**) loading pattern NM-9-2.

In order to further explore the influence of specimen size and loading pattern on the mechanical properties of aluminum alloy specimens, a total of 42 specimens are designed for the numerical analysis. The design parameters of the specimens are shown in Table 3. The loading patterns of the specimens for numerical analysis are shown in Figure 14.

### 4.3. Hysteresis Curves

**Figure 15:**
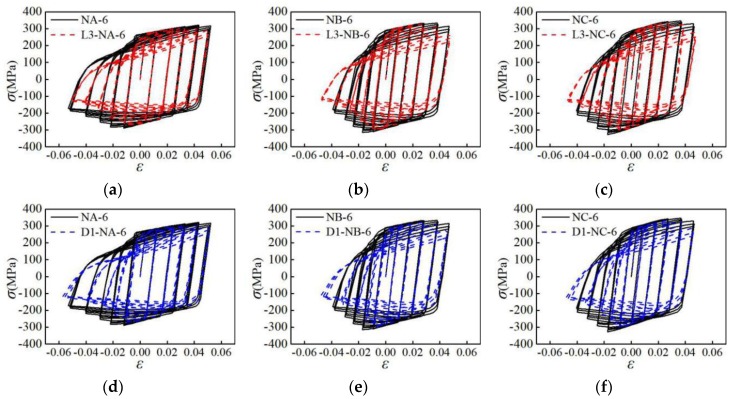
Influence of specimen size on hysteresis curves: (**a**) group A of specimens with different parallel section length; (**b**) group B of specimens with different parallel section length; (**c**) group C of specimens with different parallel section length; (**d**) group A of specimens with different parallel section diameter; (**e**) group A of specimens with different parallel section diameter; (**f**) group A of specimens with different parallel section diameter.

To investigate the influence of the specimen size on the mechanical properties, the hysteresis curves of specimens with different sizes under the NM6 loading pattern are selected, as shown in Figure 15. In Figure 15, the abscissa *ε* is the average strain of the parallel sections, and the ordinate *σ* is the average stress of the parallel sections. It can be seen from Figure 15a–c that for the NA-6, NB-6, and NC-6 specimens that have a small parallel section length, their hysteresis curves are fuller, and the degradation of curves is more gradual in the later stage of the loading. For the L3-NA-6, L3-NB-6, and L3-NC-6 specimens with larger parallel sections, the area of the hysteresis loop is relatively smaller, and the curve degradation is more obvious in the late loading stage. This indicates that increasing the parallel section length will reduce the energy consumption and plastic deformation ability of the aluminum alloy. Based on Figure 15d–f, the hysteresis curves are fuller for the NA-6, NB-6, and NC-6 specimens having larger parallel section diameters. For the D1-NA-6, D1-NB-6, and D1-NC-6 specimens with smaller parallel section diameters, the bearing capacity and stiffness degradation are more obvious in the later loading stage. It is shown that reducing the parallel section length or increasing the parallel section diameter can improve the energy-dissipating capacity and plastic deformation ability of the aluminum alloy.

### 4.4. Skeleton Curves

#### 4.4.1. Influence of Parallel Section Length

**Figure 16:**
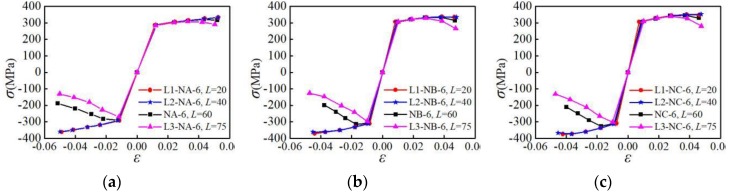
Influence of parallel section length on skeleton curves: (**a**) group A of specimens; (**b**) group B of specimens; (**c**) group C of specimens.

The skeleton curves of the specimens with different parallel section lengths subjected to the NM6 loading pattern are analyzed, as shown in Figure 16. It is indicated that for the L3-NA-6, L3-NB-6, and L3-NC-6 specimens with a large parallel section length, their rigidity and bearing capacity during reverse loading are smaller than those in the forward loading, and the degradation of skeleton curves in the later loading stage is more significant. For the L1-NA-6, L1-NB-6, and L1-NC-6 specimens with smaller parallel section lengths, the bearing capacity is larger, and the degradation of the skeleton curve in the later stage of loading is not obvious. When the parallel section length is greater than 40 mm, the peak load and envelope area of skeleton curve decrease gradually with the increasing of the parallel section length. When the parallel section length is less than 40 mm, it has only limited influence on the skeleton curve. It is shown that for all the three types of aluminum alloys, the influence of the parallel section length on the skeleton curve is significant when the parallel section length is more than 40 mm.

#### 4.4.2. Influence of Parallel Section Diameter

**Figure 17:**
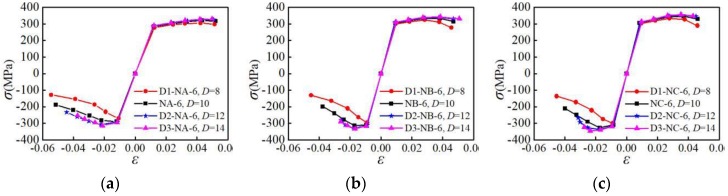
Influence of parallel section diameter on skeleton curves: (**a**) group A of specimens; (**b**) group B of specimens; (**c**) group C of specimens.

The skeleton curves of the specimens with different parallel section diameters subjected to the NM6 loading pattern are analyzed, as shown in Figure 17. As can be seen from Figure 17, for the D1-NA-6, D1-NB-6, and D1-NC-6 specimens having small parallel section diameters, the degradation of rigidity and strength are remarkable. For the D3-NA-6, D3-NB-6, and D3-NC-6 specimens with larger parallel section diameters, their bearing capacity is larger, and the skeleton curve degrades moderately. When the parallel section diameter is less than 12 mm, the peak load and envelope area of skeleton curve gradually reduce with the decrease in the parallel section diameter. When the parallel section diameter is larger than 12 mm, it has only a slight influence on the skeleton curve. It is shown that for the aluminum alloy, the parallel section diameter has a significant influence on the skeleton curve when the parallel section diameter is less than 12 mm. As the parallel section diameter decreases, the bearing capacity and energy consumption capacity decline gradually.

#### 4.4.3. Influence of Loading Pattern

**Figure 18:**
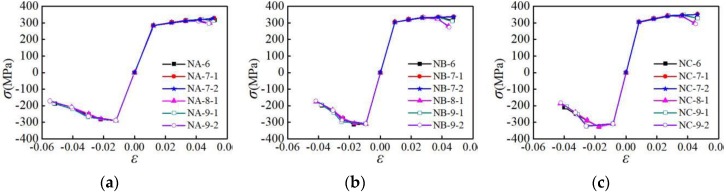
Influence of loading pattern on skeleton curves: (**a**) group A of specimens; (**b**) group B of specimens; (**c**) group C of specimens.

The skeleton curves of three different aluminum alloy specimens are analyzed under different loading patterns, as shown in Figure 18. In the repeated tension-compression loading patterns, as the number of loading cycles increases, the peak load of the skeleton curve decreases. In the repeated tensile loading pattern, the effect of the number of loading cycles on the skeleton curve is relatively small. When the initial loading strain of the repeated loading pattern is less than 2.0%, the influence of the strain amplitude in the later stage of the loading on the skeleton curve is relatively small.

### 4.5. Stiffness Degradation

In order to study the stiffness degradation law of aluminum alloys under low-cycle fatigue loading the secant stiffness is used to quantitatively analyze the stiffness degradation. The calculation formula of the secant stiffness *K*_i_ is:

(1) $$K_{i}=\frac{\left|+\sigma_{i}\right|+\left|-\sigma_{i}\right|}{\left|+\varepsilon_{i}\right|+\left|-\varepsilon_{i}\right|}$$

Where, *σ*_i_ is the peak stress of the skeleton curve at the *i*_th_ loading during low-cycle fatigue loading; *ε*_i_ is the strain value corresponding to *σ*_i_.

#### 4.5.1. Influence of Material Type

**Figure 19:**
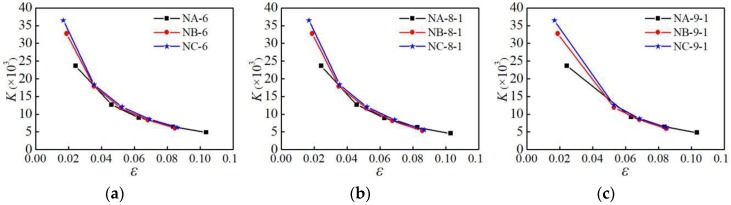
Influence of material type on secant stiffness: (**a**) loading patternNM-6; (**b**) loading pattern NM-8-1; (**c**) loading patternNM-9-1.

The secant stiffness of the three aluminum alloy specimens under the action of NM6, NM8-1, and NM9-1 loading patterns is analyzed, as shown in Figure 19. It can be seen from Figure 19 that the initial stiffness of group C is the largest, and the initial stiffness of group A is the smallest. In the initial stage of loading, the secant stiffness of the three sets of specimens degrades rapidly. In the later stage of loading, the stiffness degradation of the three specimens to slow down and tend to the same value.

#### 4.5.2. Influence of Parallel Section Length

**Figure 20:**
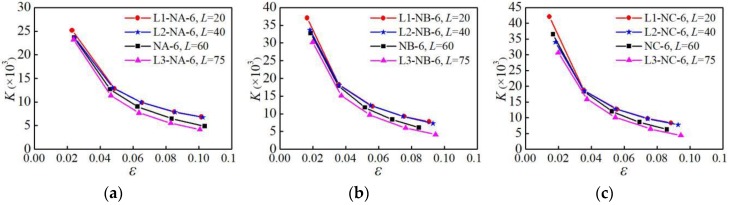
Influence of parallel section length on secant stiffness: (**a**) group A of specimens; (**b**) group B of specimens; (**c**) group C of specimens.

The secant stiffness of the specimens with different parallel section lengths under the NM6 loading pattern is analyzed, as shown in Figure 20. It can be seen from Figure 20 that when the parallel section length is greater than 40 mm, it has a significant influence on the stiffness. As the parallel section length increases, the secant stiffness gradually decreases, and the stiffness degradation is more significant.

#### 4.5.3. Influence of Parallel Section Diameter

**Figure 21:**
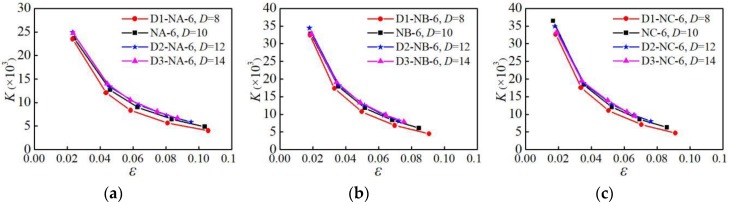
Influence of parallel section diameter on secant stiffness: (**a**) group A of specimens; (**b**) group B of specimens; (**c**) group C of specimens.

The secant stiffness of the specimens with different parallel section diameters under the NM6 loading pattern is analyzed, as shown in Figure 21. It can be seen from Figure 21 that the smaller the parallel section diameter, the more significant the degradation of the secant stiffness is. In the initial stage of loading, the stiffness of the specimen degrades faster, and the stiffness degradation in the later stage of loading becomes slower.

## 5. Strength Design Formula

Due to the complexity of the material testing and finite element simulation under low-cycle fatigue loading, it is not easy to directly estimate the tensile strength of the specimen under low-cycle fatigue loading. In order to facilitate the application of aluminum alloys in seismic engineering, based on the experimental research and numerical analysis results, this study proposes a formula for calculating the tensile strength *f*_u_ of aluminum alloys under low-cycle fatigue loading:

(2) $$\alpha (\frac{\varepsilon_{y}}{\varepsilon_{\max}})+\beta (\frac{f_{y}}{f_{u}})=1$$

where *ε*_y_ is the yield strain measured by uniaxial tension, *f*_y_ is the yield strength measured by uniaxial tension, *ε*_max_ is the maximum load strain value experienced during low-cycle fatigue loading, and *f*_u_ is the tensile capacity under low-cycle fatigue loading. *α* and *β* are parameters related to the length and diameter of the parallel section of the specimen and the number of loading cycles. Based on the given parameters of the aluminum alloy materials, 1stOpt is used to determine *α* and *β* parameters. In the repeated tensile loading pattern, *α* = (−0.000051 − 0.003035*L* − 0.000506*D* − 0.001646*N*); *β* = (2.7387 + 0.09794*L* + 0.2825*D* + 0.000075*N*). In the repeated tension-compression loading patterns, *α* = (−0.7966 + 0.1553 *L* − 0.9601 *D* + 0.08113 *N*); *β* = (11.1786 − 0.2137 *L* + 1.3808 *D* − 0.1099 *N*). *L* is the parallel section length, *D* is the parallel section diameter, and *N* is the total number of loading cycles.

**Figure 22:**
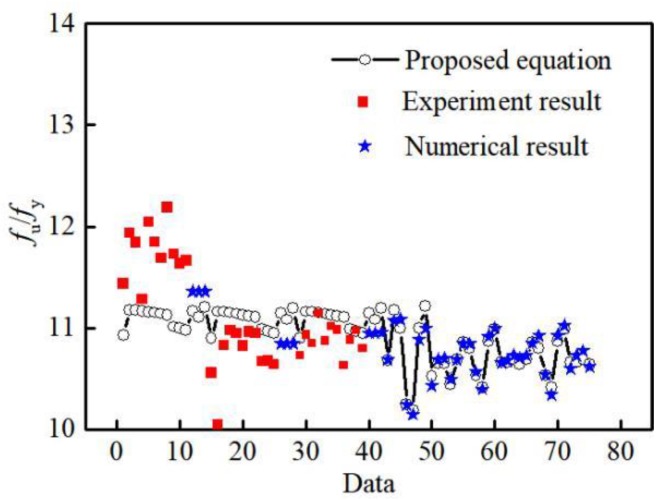
Comparison of the proposed equations with the results of test and numerical analysis.

The tensile strength *f*_u_ of the specimen under low-cycle fatigue loading is calculated using formula (2) and compared with results of the test and the finite element analysis, as shown in Figure 22. As can be seen from Figure 22, the error between the calculation formula (2) and the test result is less than 10% and that with the finite element result is less than 3.5%. Thus, it can be concluded that formula (2) has the requisite accuracy.

## 6. Conclusions

To investigate the mechanical properties of aluminum alloys under low-cycle fatigue loading, the mechanical properties of such aluminum alloys were tested under repeated tensile loads. The finite element models of aluminum alloy specimens subjected to low-cycle fatigue loading were established and compared with the experimental results. Furthermore, the mechanical properties of aluminum alloys under low-cycle fatigue loading were analyzed. The main findings of this study are as follows:

(1) When the specimen is monotonously stretched to breaking, the fracture surface is dark gray in color and has irregular fibers, and the fracture surface is rough; the fracture cross section and tensile stress direction are at an angle of 45°, which indicates a shear fracture. When the specimen is repeatedly stretched, the fracture surface is dark gray and uneven, the inner ring of the fracture cross section is perpendicular to the tensile stress, and the outer ring and tensile stress direction are at an angle of 45°. The failure of the specimen is the result of the combined action of tensile stress and plastic fatigue damage, which is a mixed fracture of tensile fracture and fatigue fracture.

(2) The AA6061, AA7075, and AA6063 aluminum alloys show cyclic softening characteristics under repeated loading. They show rapid softening in the initial stage of repeated loading, while the softening rate slows down in the later stage. 

(3) When the initial stress amplitude of repeated loading is greater than 2.5%, the repeated tensile loading has a detrimental effect on the deformability of the aluminum alloy.

(4) Compared with the repeated tensile loading pattern, the effect of the number of loading cycles on the skeleton curve is more significant in the repeated tension-compression loading pattern; and with the increase in the number of loading cycles, the bearing capacity and stiffness of the specimen will decrease.

(5) For the aluminum alloy specimens, when the parallel section length is greater than 40 mm, its influence on the skeleton curve is significant. When the parallel section diameter is less than 12 mm, the influence of the parallel section diameter on the skeleton curve is significant. Reducing the parallel section length or increasing the parallel section diameter can improve the energy dissipation capability and plastic deformation ability of the aluminum alloy.

(6) Based on the results of experimental research and numerical analysis, a formula for calculating the tensile strength of aluminum alloys under low-cycle fatigue loading is proposed.

## Figures and Tables

**Table 1:** Specimen description.

Specimen Label	Material Type	Loading Patterns	Specimen Label	Material Type	Loading Patterns
A-1	AA6061	NM1	B-4-1	AA7075	NM4-1
A-2-1	AA6061	NM2-1	B-4-2	AA7075	NM4-2
A-2-2	AA6061	NM2-2	B-4-3	AA7075	NM4-3
A-3-1	AA6061	NM3-1	B-5-1	AA7075	NM5-1
A-3-2	AA6061	NM3-2	B-5-2	AA7075	NM5-2
A-3-3	AA6061	NM3-3	B-5-3	AA7075	NM5-3
A-4-1	AA6061	NM4-1	C-1	AA6063	NM1
A-4-2	AA6061	NM4-2	C-2-1	AA6063	NM2-1
A-4-3	AA6061	NM4-3	C-2-2	AA6063	NM2-2
A-5-1	AA6061	NM5-1	C-3-1	AA6063	NM3-1
A-5-2	AA6061	NM5-2	C-3-2	AA6063	NM3-2
A-5-3	AA6061	NM5-3	C-3-3	AA6063	NM3-3
B-1	AA7075	NM1	C-4-1	AA6063	NM4-1
B-2-1	AA7075	NM2-1	C-4-2	AA6063	NM4-2

**Table 2:** Summary of results.

Specimen Label	*f*_u_ (MPa)	*ε*_u_ (%)	*E* (J)	*δ* (%)	Specimen Label	*f*_u_ (MPa)	*ε*_u_ (%)	*E* (J)	*δ* (%)
A-1	369.32	6.59%	164.55	11.98%	B-4-1	336.93	4.29%	137.80	10.90%
A-2-1	343.27	8.55%	148.75	11.78%	B-4-2	341.20	4.64%	119.55	9.36%
A-2-2	358.23	5.77%	132.23	10.62%	B-4-3	340.74	3.99%	150.23	11.68%
A-3-1	355.47	5.03%	128.81	9.68%	B-5-1	332.17	2.89%	135.43	10.80%
A-3-2	338.69	5.48%	122.95	9.60%	B-5-2	332.25	5.56%	136.22	10.70%
A-3-3	361.55	4.80%	153.34	10.74%	B-5-3	331.11	2.63%	123.83	9.72%
A-4-1	355.55	5.03%	151.32	11.98%	C-1	347.66	5.10%	128.17	9.78%
A-4-2	350.78	4.29%	159.51	12.04%	C-2-1	346.77	2.66%	101.17	7.78%
A-4-3	365.82	5.04%	153.48	10.94%	C-2-2	353.24	5.41%	116.34	8.96%
A-5-1	332.05	4.16%	123.36	9.74%	C-3-1	350.63	4.68%	134.82	10.46%
A-5-2	349.25	8.55%	164.78	11.44%	C-3-2	360.08	5.14%	141.15	10.72%
A-5-3	322.06	6.95%	145.67	12.00%	C-3-3	351.39	4.87%	80.08	6.22%
B-1	330.34	4.97%	120.08	9.80%	C-4-1	355.92	4.75%	107.59	8.76%
B-2-1	328.58	4.78%	114.15	9.16%	C-4-2	354.80	4.57%	132.77	10.44%
B-2-2	312.84	5.02%	95.22	8.66%	C-4-3	343.58	4.72%	125.53	9.74%
B-3-1	337.03	4.82%	105.88	8.44%	C-5-1	351.66	3.18%	101.90	7.96%
B-3-2	341.66	4.88%	122.85	9.64%	C-5-2	354.61	3.03%	84.08	6.74%
B-3-3	340.86	4.85%	139.84	11.06%	C-5-3	348.83	2.83%	79.74	6.70%

**Table 3:** Design of specimen for the numerical analysis.

Parallel Section Length *L*_C_ (mm)	Parallel Section Diameter (mm)	Loading Patterns	Specimen Label (Material Type)	Specimen Label (Material Type)	Specimen Label (Material Type)
60	10	NM6	NA-6 (AA6061)	NB-6 (AA7075)	NC-6 (AA6063)
20	10	NM6	L1-NA-6 (AA6061)	L1-NB-6 (AA7075)	L1-NC-6 (AA6063)
40	10	NM6	L2-NA-6 (AA6061)	L2-NB-6 (AA7075)	L2-NC-6 (AA6063)
75	10	NM6	L3-NA-6 (AA6061)	L3-NB-6 (AA7075)	L3-NC-6 (AA6063)
60	8	NM6	D1-NA-6 (AA6061)	D1-NB-6 (AA7075)	D1-NC-6 (AA6063)
60	12	NM6	D2-NA-6 (AA6061)	D2-NB-6 (AA7075)	D2-NC-6 (AA6063)
60	14	NM6	D3-NA-6 (AA6061)	D3-NB-6 (AA7075)	D3-NC-6 (AA6063)
60	10	NM7-1	NA-7-1 (AA6061)	NB-7-1 (AA7075)	NC-7-1 (AA6063)
60	10	NM7-2	NA-7-2 (AA6061)	NB-7-2 (AA7075)	NC-7-2 (AA6063)
60	10	NM7-3	NA-7-3 AA(6061)	NB-7-3 (AA7075)	NC-7-3 (AA6063)
60	10	NM8-1	NA-8-1 (AA6061)	NB-8-1 (AA7075)	NC-8-1 (AA6063)
60	10	NM8-2	NA-8-2 (AA6061)	NB-8-2 (AA7075)	NC-8-2 (AA6063)
60	10	NM9-1	NA-9-1 (AA6061)	NB-9-1 (AA7075)	NC-9-1 (AA6063)
60	10	NM9-2	NA-9-2 AA(6061)	NB-9-2 (AA7075)	NC-9-2 (AA6063)

## References

[1] Guo X., Xiong Z., Luo Y., Xu H., Liang S. (2016). Block tearing and local buckling of aluminum alloy gusset joint plates. KSCE J. Civ. Eng..

[2] Guo X., Xiong Z., Luo Y., Qiu L., Liu J. (2015). Experimental investigation on the semi-rigid behaviour of aluminium alloy gusset joints. Thin Walled Struct..

[3] Xue L. (2008). A unified expression for low cycle fatigue and extremely low cycle fatigue and its implication for monotonic loading. Int. J. Fatigue.

[4] Libertiny G.Z. (1967). Effect of Hydrostatic Pressure on the Short Life Fatigue Property of an Alloy Steel. Proc. Inst. Mech. Eng..

[5] Shaha S.K., Czerwinski F., Kasprzak W., Friedman J., Chen D.L. (2015). Improving High-Temperature Tensile and Low-Cycle Fatigue Behavior of Al-Si-Cu-Mg Alloys Through Micro-additions of Ti, V, and Zr. Metall. Mater. Trans. A.

[6] Huang H., Dong Y., Xing Y., Jia Z., Liu Q. (2018). Low cycle fatigue behaviour at 300 °C and microstructure of Al-Si-Mg casting alloys with Zr and Hf additions. J. Alloys Compd..

[7] Liu H.J., Fujii H., Maeda M., Nogi K. (2003). Tensile properties and fracture locations of friction-stir-welded joints of 2017-T351 aluminum alloy. J. Mater. Process. Technol..

[8] Lee W.B., Yeon Y.M., Jung S.B. (2003). The improvement of mechanical properties of friction-stir-welded A356 Al alloy. Mater. Sci. Eng. A.

[9] Srivatsan T.S. (1991). The low-cycle fatigue and cyclic fracture behaviour of 7150 aluminium alloy. Int. J. Fatigue.

[10] Hao H., Ye D., Chen C. (2014). Strain ratio effects on low-cycle fatigue behavior and deformation microstructure of 2124-T851 aluminum alloy. Mater. Sci. Eng. A.

[11] Arcari A., De Vita R., Dowling N.E. (2009). Mean stress relaxation during cyclic straining of high strength aluminum alloys. Int. J. Fatigue.

[12] Arcari A., Dowling N.E. (2012). Modeling mean stress relaxation in variable amplitude loading for 7075-T6511 and 7249-T76511 high strength aluminum alloys. Int. J. Fatigue.

[13] Kim S., Burns J.T., Gangloff R.P. (2009). Fatigue crack formation and growth from localized corrosion in Al–Zn–Mg–Cu. Eng. Fract. Mech..

[14] Lin C., Yang S. (1998). Corrosion fatigue behavior of 7050 aluminum alloys in different tempers. Eng. Fract. Mech..

[15] Burns J.T., Gupta V.K., Agnew S.R., Gangloff R.P. (2013). Effect of low temperature on fatigue crack formation and microstructure-scale propagation in legacy and modern Al–Zn–Mg–Cu alloys. Int. J. Fatigue.

[16] Gasquères C., Sarrazin-Baudoux C., Petit J., Dumont D. (2005). Fatigue crack propagation in an aluminium alloy at 223K. Scr. Mater..

[17] Conley J.G., Huang J., Asada J., Akiba K. (2000). Modeling the effects of cooling rate, hydrogen content, grain refiner and modifier on microporosity formation in Al A356 alloys. Mater. Sci. Eng. A.

[18] Wang Q.G., Apelian D., Lados D.A. (2001). Fatigue behavior of A356-T6 aluminum cast alloys. Part I. Effect of casting defects. J. Light Met..

[19] Azadi M., Shirazabad M.M. (2013). Heat treatment effect on thermo-mechanical fatigue and low cycle fatigue behaviors of A356.0 aluminum alloy. Mater. Des..

[20] Zhu M., Jian Z., Yang G., Zhou Y. (2012). Effects of T6 heat treatment on the microstructure, tensile properties, and fracture behavior of the modified A356 alloys. Mater. Des..

[21] Wang C., Usami T., Funayama J., Imase F. (2013). Low-cycle fatigue testing of extruded aluminium alloy buckling-restrained braces. Eng. Struct..

[22] Underhill P.R., DuQuesnay D.L. (2009). Effect of small cycles and load spectrum truncation on the fatigue life scatter in 7050 Al alloy. Int. J. Fatigue.

[23] DuQuesnay D.L., Underhill P.R. (2010). Fatigue life scatter in 7xxx series aluminum alloys. Int. J. Fatigue.

[24] Zhang L., Wang G., Cheng J., Jiang L. (2003). Investigation of the low-cycle fatigue life under multi-axial non-proportional loading. Mater. Sci. Eng. A.

[25] (2016). ISO 6892-1: 2016 Metallic materials—Tensile testing—Part 1: Method of test at room temperature.

